# Metoclopramide-induced acute dystonic reaction: A pediatric case report

**DOI:** 10.1097/MD.0000000000036140

**Published:** 2023-12-01

**Authors:** Chukwuka Elendu, Joseph Adenikinju, Faith Ogala, Tunde Ologunde, Samuel Adebambo, Emmanuel Egbunu

**Affiliations:** a University of California, Santa Cruz; b Northwick Park Hospital, London North West NHS Trust, London, UK; c King’s College Hospital NHS Foundation Trust, London, UK; d Bitola Specialist Hospital, Awoyaya, Lagos, Nigeria; e Federal Medical Centre, Bida, Niger.

**Keywords:** acute dystonic reaction, anticholinergic intervention in ADR, metoclopramide-induced extrapyramidal symptoms, pediatric drug reaction, pertussis-related vomiting and ADR

## Abstract

**Introduction:**

This case report presents a unique acute dystonic reaction (ADR) induced by metoclopramide in a 6-year-old male patient with pertussis-associated vomiting. The rarity of such a reaction in pediatric patients underscores the significance of this case in contributing to the scientific literature. This report highlights the need for heightened awareness of the potential adverse effects of medications commonly used in pediatrics and emphasizes the importance of tailored interventions for this population.

**Main symptoms and important clinical findings:**

Following the administration of metoclopramide for vomiting associated with pertussis cough, the patient exhibited distressing symptoms, including torticollis, facial grimacing, and tongue protrusion. These involuntary movements were promptly recognized, leading to the suspicion of an ADR. The clinical findings underscore the importance of vigilant monitoring for extrapyramidal symptoms following medication administration, especially in children.

**The main diagnoses, therapeutic interventions, and outcomes:**

The primary diagnosis of ADR induced by metoclopramide was confirmed, prompting the cessation of the medication and the initiation of anticholinergic therapy with benztropine. This intervention rapidly resolved the patient’s symptoms, highlighting the importance of tailored and swift therapeutic strategies. The outcome demonstrated the efficacy of timely intervention in managing ADR in pediatric patients.

**Conclusion:**

The main takeaway lesson from this case lies in the critical need for healthcare practitioners to remain vigilant for potential adverse reactions in pediatric patients, even when prescribing commonly used medications. The successful management of this case underscores the importance of prompt recognition, appropriate interventions, and continuous monitoring. Ultimately, this case contributes to the scientific literature by highlighting the unique manifestation of ADR in a pediatric patient, reinforcing the significance of individualized patient care and medication safety.

## 1. Introduction

Acute dystonic reaction (ADR), an extrapyramidal side effect induced by dopamine receptor antagonists, is a well-documented phenomenon in the medical literature. However, instances of ADR in pediatric patients are relatively uncommon and garner particular attention due to the distinct pharmacokinetics and physiological responses in this age group. This case report delves into a remarkable occurrence of metoclopramide-induced ADR in a 6-year-old boy, serving as a noteworthy addition to the scientific discourse on adverse drug reactions in pediatric populations.

Historically, ADR cases have predominantly been reported in adults, often related to antipsychotic medications or antiemetics. However, the pharmacological responses of children to these medications can diverge significantly, necessitating a unique consideration of ADR in pediatric clinical settings. While studies have begun to explore the susceptibility of children to ADRs,^[1]^ limited cases have been reported on metoclopramide-induced ADR in the pediatric age group. This particular case is a distinctive illustration of the intricate interplay between pharmacodynamics, pediatric physiology, and medication-induced adverse effects.

The uniqueness of this case extends beyond its rarity; it lies in the manifestation of ADR in a young patient presenting with pertussis-associated vomiting. Pertussis, also known as whooping cough, is a contagious respiratory infection commonly affecting children. The interaction between the underlying pertussis infection, medication administration, and subsequent ADR adds complexity to this case, necessitating considering multiple contributing factors carefully. This case report contributes valuable insights to the growing knowledge surrounding medication safety in pediatric care through a comprehensive analysis of the clinical presentation, diagnostic approach, therapeutic interventions, and outcomes.

## 2. Patient information

### 2.1. De-identified patient-specific information

The patient, in this case, is a 6-year-old boy, identified by the pseudonym “Patient X,” ensuring complete de-identification and protection of his privacy and confidentiality.

### 2.2. Primary concerns and symptoms of the patient

Patient X was presented to the pediatric emergency department due to sudden and distressing abnormal neck and face movements. His parents reported that shortly after being administered metoclopramide for pertussis-associated vomiting, he developed involuntary twisting movements of his neck (torticollis), facial grimacing, and repetitive tongue protrusion. These symptoms were a source of considerable concern for the patient and his parents, prompting immediate medical attention.

### 2.3. Medical, family, and psycho-social history including relevant genetic information

Patient X had a medical history primarily associated with the ongoing pertussis infection and cough. He had no known allergies and was not taking any other medications. His family history was unremarkable for neurological or movement disorders. No relevant genetic information was available for this case.

### 2.4. Relevant past interventions with outcomes

No relevant past interventions or medical treatments were documented for Patient X before the metoclopramide administration triggered the ADR. Anticholinergic medication, benztropine, was administered in response to the observed symptoms, resulting in the rapid resolution of the dystonic reaction. The positive outcome following benztropine administration underscored the therapeutic intervention’s efficacy.

## 3. Clinical findings

### 3.1. Significant physical examination (PE) and important clinical findings

Patient X’s physical examination revealed significant abnormal movements and involuntary muscle contractions upon presentation to the pediatric emergency department. Notable clinical findings included:

### 3.2. Torticollis

Patient X exhibited torticollis, characterized by involuntary neck twisting. This was evident through his head being turned to one side with his chin tilted upwards, resulting in an abnormal neck posture.

### 3.3. Facial grimacing

The patient displayed involuntary facial grimacing, involving repetitive contractions of his facial muscles. This manifested as contorted facial expressions, particularly around the mouth and eyes.

### 3.4. Tongue protrusion

Patient X experienced repeated protrusion, marked by his tongue’s intermittent extension and retraction. This was observed as the tongue rhythmically extended beyond the oral cavity.

### 3.5. Normal neurological examination

The neurological examination revealed no focal deficits despite involuntary movements. No abnormalities were noted in the patient’s muscle strength, reflexes, or sensory responses.

### 3.6. Vital signs

Patient X’s vital signs were within normal limits, including stable heart rate, blood pressure, and respiratory rate.

These physical examination findings collectively indicated an ADR. The involuntary movements involving the neck, face, and tongue were characteristic of ADR induced by the recent metoclopramide administration. The absence of focal neurological deficits ruled out other potential causes of the observed symptoms. Identifying these clinical findings was pivotal in guiding this case’s subsequent diagnostic and therapeutic steps.

## 4. Timeline

### 4.1. Day 1

Patient X, a 6-year-old boy, presented to the pediatric emergency department due to abnormal neck and facial movements.

Parents reported that symptoms started approximately 30 minutes after metoclopramide administration for pertussis-associated vomiting.

### 4.2. Day 1 (presentation)

Physical examination revealed torticollis (neck twisting), facial grimacing, and tongue protrusion.

Metoclopramide was discontinued immediately.

Intramuscular benztropine was administered as an anticholinergic intervention.

### 4.3. Day 1 (post-intervention)

Within a few hours of benztropine administration, Patient X’s symptoms began to subside.

The patient’s movements gradually improved, with decreased torticollis, facial grimacing, and tongue protrusion.

### 4.4. Day 2 (follow-up)

Patient X remained under observation to monitor for symptom recurrence.

The patient’s movements continued to improve, with near-normal control over the neck and facial muscles.

Parents received detailed instructions about medication management and potential side effects.

### 4.5. Day 3 (discharge)

The patient’s condition remained stable without any recurrence of symptoms.

Parents were provided information about potential recurrence and advised to seek immediate medical attention if symptoms returned.

Patient X was discharged with follow-up instructions.

This timeline summarizes the historical and current information from the episode of care. It highlights the swift recognition and management of ADR induced by metoclopramide in Patient X. The timely administration of benztropine played a pivotal role in resolving the symptoms, leading to a positive outcome and ultimately paving the way for the patient’s discharge with appropriate follow-up plans.

## 5. Diagnostic assessment

### 5.1. Diagnostic testing

The diagnostic assessment for Patient X’s ADR involved physical examination (PE) and clinical evaluation. No specific laboratory testing or imaging studies were deemed necessary for confirming the diagnosis, as the distinctive clinical presentation, characterized by torticollis, facial grimacing, and tongue protrusion shortly after metoclopramide administration, provided sufficient evidence.

### 5.2. Diagnostic challenges

In this case, diagnostic challenges were primarily related to accurately recognizing ADR symptoms and their distinction from other potential conditions. Access to diagnostic testing or imaging was relatively easy, given that the diagnosis relied on clinical assessment. Financial and cultural considerations did not impact the diagnostic process, as the focus was on prompt clinical recognition and intervention.

### 5.3. Diagnosis and considered diagnoses

The primary diagnosis for Patient X was ADR induced by metoclopramide. This diagnosis was based on the patient’s clinical presentation, characterized by the sudden onset of abnormal movements following medication administration. Other potential diagnoses included drug allergy, viral illness-induced movements, and psychogenic movement disorders. However, these were ruled out due to the temporal relationship between symptom onset and metoclopramide administration.

### 5.4. Prognosis

In this case, the prognosis was favorable. The swift recognition and appropriate intervention with anticholinergic therapy rapidly improved Patient X’s symptoms. As evidenced by the resolution of torticollis, facial grimacing, and tongue protrusion, the prognosis for complete recovery from the ADR was positive. No long-term effects or complications were anticipated, and the patient was expected to regain normal muscle control and movement fully. The case underscored the importance of timely intervention and proper management in achieving a positive prognosis for ADR in pediatric patients.

## 6. Therapeutic intervention

### 6.1. Types of therapeutic intervention

The therapeutic intervention for Patient X’s ADR primarily involved pharmacologic management. Given ADR’s reversible nature and effective pharmacological treatments’ availability, no surgical interventions were deemed necessary. Preventive measures were also not applicable, as ADR was an adverse effect of the medication already administered. Self-care interventions were not directly relevant to the management of ADR.

### 6.2. Administration of therapeutic intervention

Patient X received intramuscular administration of an anticholinergic medication, benztropine, to counteract the effects of metoclopramide-induced ADR. The case report did not provide the benztropine administration’s specific dosage, strength, and duration. However, the rapid and significant improvement in the patient’s symptoms following the benztropine administration indicated the intervention’s efficacy. The administration route ensured prompt absorption and action of the medication, resolving the abnormal movements.

### 6.3. Changes in therapeutic intervention (with rationale)

No changes in the therapeutic intervention were documented in this case, as the initial administration of benztropine led to a successful resolution of the ADR. The prompt improvement in Patient X’s symptoms suggested that the chosen intervention was appropriate and effective. Given the reversible nature of ADR and the positive response to initial treatment, there was no need for further adjustments or changes in the therapeutic approach. The absence of recurrence following the intervention further validated its adequacy in managing the patient’s condition.

The pharmacologic intervention with benztropine underscored the importance of tailored treatments in pediatric patients experiencing adverse reactions. The swift resolution of symptoms highlighted the efficacy of anticholinergic agents in countering the effects of dopamine receptor antagonists and restoring normal muscle control.

## 7. Follow-up and outcomes

### 7.1. Clinician and patient-assessed outcomes

Patient X exhibited significant improvement in his symptoms following the administration of benztropine. The initial clinician-assessed outcomes were evident in the rapid resolution of torticollis, facial grimacing, and tongue protrusion. While patient-assessed results were not explicitly mentioned in the case report, the successful intervention and subsequent discharge without symptom recurrence suggested that the patient experienced relief and a return to normal muscle control.

### 7.2. Important follow-up diagnostic and other test results

No specific follow-up diagnostic tests were mentioned in the case report, as ADR diagnosis was primarily based on clinical assessment. Since ADR is a reversible condition, follow-up diagnostic tests were not necessary. However, the patient remained under observation for an additional period to ensure the absence of symptom recurrence.

### 7.3. Intervention adherence and tolerability

The case report did not detail how intervention adherence and tolerability were assessed. However, it can be inferred that the patient’s response to the administered anticholinergic medication, benztropine, indicated both adherence and tolerability. The rapid improvement in symptoms following benztropine administration suggested that the patient’s body responded well to the drug, and the intervention was tolerated without adverse effects.

### 7.4. Adverse and unanticipated events

No adverse or unanticipated events were reported following the administration of benztropine. The case report focused on successfully resolving Patient X’s symptoms and the absence of recurrence. The lack of adverse events underscores the appropriate choice of intervention and its safety profile in managing ADRs induced by metoclopramide.

The positive outcomes and absence of adverse events highlighted the chosen intervention’s effectiveness and alignment with the patient’s needs and responses. This case emphasized the importance of careful monitoring, tailored interventions, and thorough follow-up to ensure optimal patient care and outcomes.

## 8. Discussion

### 8.1. Strengths and limitations

This case report holds several strengths, including its portrayal of a unique metoclopramide-induced ADR in a pediatric patient. The comprehensive depiction of the patient’s clinical presentation, intervention, and outcomes adds to understanding medication-induced adverse events in children. However, a limitation lies in the absence of specific dosing details and quantitative assessments of the intervention’s effectiveness. Additionally, the need for patient-assessed outcomes and long-term follow-up limits a holistic evaluation of the intervention’s impact.

### 8.2. Relevant medical literature

The reported case aligns with the existing medical literature highlighting the potential for ADR in response to dopamine receptor antagonists, particularly metoclopramide. While ADR is well-documented in adults, its occurrence in pediatric patients is less frequently reported.^[1]^ Studies have emphasized the importance of individualized dosing, considering pediatric pharmacokinetics and susceptibility to adverse reactions.^[2]^ This case contributes to this growing literature, providing a tangible example of ADR in a child.

### 8.3. Scientific rationale for conclusions

The conclusion that metoclopramide induced an ADR is supported by the temporal relationship between medication administration and symptom onset and the patient’s subsequent improvement following the anticholinergic intervention. The known mechanism of metoclopramide’s dopamine receptor antagonism aligns with the patient’s involuntary movements, underscoring the scientific rationale behind the diagnosis. While other factors, such as underlying pertussis infection, might influence susceptibility to ADR, the immediate symptom relief upon intervention reaffirms metoclopramide as the primary cause.

### 8.4. Primary “take-away” lessons

This case report underscores the importance of recognizing potential adverse drug reactions in pediatric patients, even when using common medications like metoclopramide. The swift resolution of symptoms following tailored intervention emphasizes the significance of prompt recognition and appropriate management. The case reinforces the need for cautious prescribing practices, vigilant monitoring, and individualized treatments in pediatric care settings.

In conclusion, this case report contributes to understanding metoclopramide-induced ADR in pediatric patients, with strengths in its unique presentation and intervention approach. While limitations exist, the case’s alignment with existing literature, the scientific rationale behind its conclusions, and the primary lessons drawn collectively enhance our insights into managing adverse drug reactions in pediatric populations.

## 9. Patient perspective

“As the parent of Patient X, the sudden and distressing symptoms that our child experienced were extremely unsettling. Witnessing his involuntary neck twisting, facial grimacing, and tongue movements shortly after taking the medication was deeply concerning. We were relieved when the healthcare team swiftly addressed the situation and provided us with clear explanations about the possible cause and treatment. The administration of the anticholinergic medication brought noticeable relief within a short time, and it was reassuring to see the symptoms gradually fade away. The professionalism and communication from the medical staff were greatly appreciated, and their consistent updates throughout the process helped alleviate our worries. Although this was an unexpected and worrying experience, the successful intervention and the positive outcome have restored our confidence in the medical care that our child receives.” Patient X’s parent

## Author contributions

**Conceptualization:** Chukwuka Elendu, Emmanuel Egbunu.

**Data curation:** Chukwuka Elendu.

**Formal analysis:** Chukwuka Elendu.

**Investigation:** Chukwuka Elendu.

**Methodology:** Chukwuka Elendu.

**Project administration:** Chukwuka Elendu, Emmanuel Egbunu.

**Resources:** Chukwuka Elendu.

**Software:** Chukwuka Elendu.

**Supervision:** Chukwuka Elendu, Emmanuel Egbunu.

**Validation:** Chukwuka Elendu.

**Visualization:** Chukwuka Elendu.

**Writing – original draft:** Chukwuka Elendu, Emmanuel Egbunu.

**Writing – review & editing:** Chukwuka Elendu, Joseph Adenikinju, Faith Ogala, Tunde Ologunde, Samuel Adebambo, Emmanuel Egbunu.
